# Sinigrin Enhanced Antiasthmatic Effects of Beta Adrenergic Receptors Agonists by Regulating cAMP-Mediated Pathways

**DOI:** 10.3389/fphar.2020.00723

**Published:** 2020-05-20

**Authors:** Simeng Chu, Wenjuan Liu, Yujie Lu, Menglin Yan, Yingying Guo, Nianwei Chang, Min Jiang, Gang Bai

**Affiliations:** ^1^State Key Laboratory of Medicinal Chemical Biology, College of Pharmacy and Tianjin Key Laboratory of Molecular Drug Research, Nankai University, Tianjin, China; ^2^Graduate School of Tianjin University of Traditional Chinese Medicine, Tianjin, China

**Keywords:** asthma, PDE4 inhibitor, sinigrin, airway smooth muscle, beta-adrenergic receptor agonists

## Abstract

Millions of patients suffer from asthma worldwide. However, the first-line drugs used to treat asthma, namely, the beta-adrenergic receptors agonists (β-agonists), are not recommended for use as monotherapy because of their severe dose-related side effects. This limitation has prompted the search for new therapies, which can be used in conjunction with β--agonists so that lower doses can be administered. Sinigrin is a major compound found in many antiasthmatic medicinal plants. In this study, we explored the antiasthmatic activity of sinigrin when used in combination with β-agonists and its underlying mechanism. Sinigrin enhanced the asthma-relieving effects of isoproterenol and reduced the effective isoproterenol dose in an acute-asthma model in guinea pigs. Mechanistically, sinigrin enhanced the cAMP levels induced by β-agonists by inhibiting PDE4. The resulting increase in cAMP levels stimulated the activity of the downstream effector protein kinase A, which would be expected to ultimately induce the relaxation of airway smooth muscle. In conclusion, sinigrin enhances the asthma-relieving effects of β-agonists by regulating the cAMP signaling pathway and represents a potential add-on drug to β-agonists for the treatment of asthma.

## Introduction

Asthma is a chronic inflammatory airway disease (Lowhagen, 2015) that affects millions of patients worldwide. The significant financial burden caused by treatment significantly reduces the quality of life for patients with asthma (Castillo et al., 2017; Huang et al., 2019; Brusselle and Ko, 2019). Currently, asthma therapy includes the use of inhalable corticosteroids (ICSs), anticholinergic drugs, and beta-2 adrenergic receptor agonists (BAs) (Lommatzsch and Virchow, 2014; Castillo et al., 2017; Suissa et al., 2017). BAs continue to be widely prescribed to provide rapid, as-needed, relief of symptoms and are always used as an emergency medication for clinical treatment, although they have severe cardiovascular adverse effects including increasing heart rate, palpitations, vasodilation, arrhythmia, and reflex tachycardia (Cazzola et al., 2005; Huang et al., 2019). Other side effects include ketoacidosis (Philipson, 2002), hypokalemia (Scheinin et al., 1987), and dose-related tremors (Cazzola et al., 2005). Those adverse effects can lead to an increased risk of exacerbating asthma. As a result, long-acting beta-2 adrenergic receptor agonists are not recommended for use in monotherapy for the treatment of asthma (Al-Sajee et al., 2019).

One of the main factors that cause side effects is inappropriate dosing. Hence, reducing the effective clinical dose for BAs may be of benefit to address this problem. Currently, oral PDE4 inhibitors are used as an add-on therapy for treating chronic respiratory diseases, including asthma and chronic obstructive pulmonary disease (COPD) (Parikh and Chakraborti, 2016; Zhang et al., 2018; Al-Sajee et al., 2019; Zuo et al., 2019). The discovery of newer PDE4 inhibitors, which can reduce the effective dose of BAs, may therefore be of benefit in reducing the possibility of adverse effects in patients with asthma.

2-propenylglucosinolate (sinigrin, SG) is a natural aliphatic glucoside that is found in the seeds of *Brassica* plants including many cough relieving and antiasthmatic traditional Chinese medicines (TCMs) such as *Descurainiae Semen* (Baek et al., 2018), *Raphani Semen* (Sham et al., 2013), and *Sinapis Semen* (Liu et al., 2003). As a major component of these TCMs, many pharmacological activities for SG have been identified such as anticancer (Jie et al., 2014), antiinflammatory (Lee et al., 2017; Lee et al., 2018), wound healing (Mazumder et al., 2016), and antiadipogenic (Lee et al., 2018) properties. However, the antiasthmatic effects of SG remain unclear. In this study, we set out to explore the antiasthmatic activity of SG and its mechanism of action.

## Material and Methods

### Chemicals and Reagents

(-)-Sinigrin hydrate (SG, sigma, 99.0%) was purchased from Beijing Yinuokai Technology Co., Ltd. (Beijing, China). Rolipram (rol, 98%) was obtained from 3AChem (Shanghai, China). Isoproterenol hydrochloride (Iso), aminophylline hydrate (Ami), acetylcholine (Ach), propranolol hydrochloride (Pro), salbutamol (Sal), and histamine (His) were purchased from Beijing Solarbio Science & Technology Co., Ltd. (Beijing, China). The cAMP luciferase reporter plasmid pGL4.29 and Renilla luciferase reporter vector plasmid pRL-TK were purchased from Promega (Madison, WI, USA). Forskolin was purchased from MCE (Milan, Italy). Dulbecco's Modified Eagle Medium (DMEM) and Dulbecco's Modified Eagle Media: Nutrient Mixture F-12 (DMEM/F-12) was purchased from LABBIOTECH Co., Ltd. (Tianjin, China).

### Cell Culture

The Chinese hamster ovary (CHO) cell line overexpressing the beta-2 adrenergic receptor (β_2_AR-CHO) was constructed in our laboratory. Cells were cultured in DMEM/F-12 medium supplemented with 1% penicillin/streptomycin, 10% fetal bovine serum (FBS), and 0.8% G-418.

Human bronchial smooth muscle cells (HBSMCs) were purchased from the American Type Culture Collection (ATCC) and cultured in DMEM containing 10% FBS, 100 µg/ml penicillin, and 100 µg/ml streptomycin.

Both cell lines were maintained at 37°C in a humidified atmosphere with 5% CO_2_ and passaged at regular intervals.

### Animals

Dunkin Hartley guinea pigs (250–300 g) were purchased from the Xinglong Experimental Animal Farm (Beijing, China). They were housed under a constant temperature and a controlled light/dark cycle (lights on between 7:30 and 19:30). Food and water were available *ad libitum*. All animal experiments were performed in accordance with the National Institutes of Health Guide for the Care and Use of Laboratory Animals and were approved by the Tianjin University of Traditional Chinese Medicine of Laboratory Animals Care and Use Committee (TCM-LAEC20170047).

### Determination of the Antiasthmatic Effects in Guinea Pigs Treated With Isoproterenol Combined with Sinigrin

Male guinea-pigs (n = 6) were placed under a bell cover (4 L) and then exposed for 25 s to an aerosol containing 2% acetylcholine and 0.1% w/v histamine produced by a nebulizer at a constant flow-rate of 0.25 ml/min. The animals were then divided into a control group sprayed with normal saline and the experimental groups sprayed, respectively, with isoproterenol (8 nmol/kg), isoproterenol (16 nmol/kg), aminophylline alone (2 μmol/kg), aminophylline (2 μmol/kg) mixed with isoproterenol (8 nmol/kg), SG (5, 10, 20 μmol/kg) alone, and SG (5, 10, 20 μmol/kg) mixed with isoproterenol (8 nmol/kg). The spaying was performed for 1 min, and the substances were allowed to be inhaled for 3 min before acetylcholine-histamine-aerosol exposure. The time from when the asphyxia response occurred until the guinea pig began to choke was measured as the latency time (LT, s). If a guinea pig did not begin to choke within 6 min, an LT of 360 s was assigned (Liu et al., 2008).

### Determination of cAMP Levels in β_2_AR-CHO Cells and HBSMCs

#### SG in Combination With Salbutamol

β_2_AR-CHO cells were cultured in a 96-well plate for 12 h and then transfected with the cyclic AMP CRE-Luc reporter plasmid pGL4.29 and the Renilla luciferase reporter vector plasmid pRL-TK (Promega). All cells, with the exception of the control cells, were treated with salbutamol (10^-9^ mol/L) to elevate cAMP levels. Propranolol hydrochloride (pro, 10^-5^ mol/L) was used to block effects of salbutamol and different concentrations of SG (10^-8^, 10^-7^, 10^-6^, 10^-5^ mol/L) were used in combination with salbutamol. After treating with the different drugs for 6 h, cAMP levels were measured using the Dual-Luciferase Reporter Assay System (Promega, USA).

#### SG in Combination With Isoproterenol Hydrochloride

β_2_AR-CHO cells were cultured and transfected as described above. Cells were treated with isoproterenol, SG, or rolipram (at concentrations of 10^-10^, 10^-9^, 10^-8^, 10^-7^, 10^-6^, 10^-5^, and 10^-4^ mol/L) or incubated with different concentrations of isoproterenol in combination with SG (10^-5^ mol/L) or rolipram (10^-5^ mol/L). After 6 h of treatment, cAMP levels were determined using the Dual-Luciferase Reporter Assay System (Promega, USA).

#### Quantitative Detection of cAMP Level Using a cAMP ELISA Kit

To quantitatively measure the intracellular cAMP levels in HBSMCs, the cells were seeded in a 12-well plate. The cells were incubated with rolipram (10 μmol/L), isoproterenol (10 μmol/L), rolipram (10 μmol/L) combined with isoproterenol (10 μmol/L), and SG (10 μmol/L) combined with isoproterenol (10 μmol/L) respectively for 24 h. As control, cells were incubated with DEME. Intracellular cAMP levels were determined using a cAMP ELISA Kit (JYM, Wuhan, China).

### Prediction of Potential Targets and Molecular Docking

#### Prediction of Potential Targets

The 3D structure of SG (mol.2 format) was imported into Pharm Mapper Database (http://www.lilab-ecust.cn/pharmmapper/) to predict its potential targets by reverse docking. Subsequently, asthma-related proteins were screened to predict functional protein association networks using the String 11.0 database (https://string-db.org/) and the Kyoto Encyclopedia of Genes and Genomes (KEGG, https://www.kegg.jp/).

#### Molecular Docking

The 3D structure of SG was determined in the mol.2 format using the ChemBio3D Ultra 14.0 software (PerkinElmer Inc., San Diego, CA, USA). To assess the interaction between PDE4 and SG, molecular docking was performed using the Auto Dock 4.2 software (Olson Laboratory, La Jolla, CA, USA). The crystal structure of PDE4 (PDB:5k1i) was obtained from the Protein Data Bank (http://www.rcsb.org/pdb).

### Enzyme Activity

#### Adenylyl Cyclase Activity Assay

HBSMCs were cultured in 100 mm cell culture dishes for 24 h and then divided into five groups: namely control, forskolin (50 μmol/L) and 0.1, 1, and 10 μmol/L SG groups. The cells were then incubated with forskolin or the different concentrations of SG for 12 h, and membrane proteins were collected using a cell membrane protein extraction kit (Sangon Biotech, Shanghai, China). The enzymatic activity of adenylyl cyclase (AC) was determined using a commercially available kit (Shanghai Hu Yu Biological Technology Co., Ltd., Shanghai, China) as described in the **Supplementary Material**.

Briefly, samples were added to micro-well coated with a specific anti-AC antibody (peptides A Cyclase 1α and A Cyclase 2α were synthesized based on the sequences of the catalytic site in AC (amino acids 200-280) and used to prepare the specific anti-AC antibody) and then incubated at 37°C for 30 min. The wells were then incubated with a horseradish peroxidase (HRP)-labeled-AC-antibody at 37°C for 30 min to form an antibody-antigen-enzyme-labeled antibody complex. After washing, the 3,3′,5,5′-tetramethylbenzidine (TMB) substrate was added. The TMB substrate is converted by HRP to a blue colored product and then finally to a yellow colored product in the presence of acid. The optical density (OD) at 450 nm was then measured and used to calculate the enzymatic activity according to the standard curve provided with the kit.

#### Phosphodiesterase 4 Activity Assay

HBSMCs were cultured in 100 mm dishes for 24 h, washed, and then lysed. The lysate was divided into control samples, samples treated with rolipram (10^-9^, 10^-8^, 10^-7^, 10^-6^, and 10^-5^ mol/L), and samples treated with SG (10^-9^, 10^-8^, 10^-7^, 10^-6^, and 10^-5^ mol/L) respectively. The samples were incubated for 12 h at 37°C on a shaker. The concentration of free phosphate (Pi), generated as a result of the two-step-reaction catalyzed by phosphodiesterase 4 (PDE4) and 5′-nucleotidase, was determined by measuring the absorbance at 660 nm following the instructions provided with the kit (details of the detection are provided in the **Supplementary Materials**).

#### Protein Kinase A Activity Assay

HBSMCs were divided into seven groups (n = 3), and incubated for 24 h with isoproterenol (10 μmol/L), rolipram (10 μmol/L), SG (10 μmol/L), rolipram (10 μmol/L) combined with isoproterenol (10 μmol/L), and SG (10 μmol/L) combined with isoproterenol (10 μmol/L) respectively. The control group was incubated with DMEM, and active protein kinase A (A-PKA, a recombinant full -length human (PKAcβ) produced by baculovirus infection of Sf 9 insect cells) was used as the positive control. Sample preparation and enzyme activity assay were performed according to the instructions provided with the PKA Kinase Activity Assay Kit (ab139435, Abcam, Cambridge, UK).

Briefly, first, prepared the active PKA according to instructions. Then samples were diluted with kinase dilution buffer before added into micro-well. ATP was added to initiate the kinase reaction. The wells were incubated for 90 min at 30°C before a phospho-specific antibody (obtained from the kit) was added to each well and incubated for 60 min at room temperature (RT). An HRP-conjugated secondary antibody was then added to the wells and they were incubated for 30 min at RT after which the wells were washed. The TMB substrate was then added and the well incubated for 60 min at RT. After addition of the stop solution, the OD at 450 nm was determined and used to calculate the PKA activity.

### Statistical Analysis

All data are expressed as means ± SD. Statistical analyses were performed using Graph Pad (verion5.0; Graph Pad Software, Inc., San Diego, CA, USA). A *p* < 0.05 was considered statistically significant.

## Results

### Isoproterenol Combined With SG Relieves Asthma Symptoms in Guinea Pigs

The process of drug inhalation localizes the effect of drug to lung tissue, in particular concentrating the therapeutic effect on the airway smooth muscles, while minimizing distribution of the drug to the systemic circulation. Based on these advantages we elected to use inhalation as the means of drug delivery in our animal experiment. Although there are selective PDE4 inhibitors for treating asthma, such as roflumilast, the poor solubility in normal saline makes spray administration impossible, hence aminophylline, a nonselective phosphodiesterase inhibitor, was selected as the positive control (Campbell et al., 1977; Myou et al., 2003; Hsu and Bajaj, 2020; Zafar and Zulfiqar, 2020).

**Figure 1:**
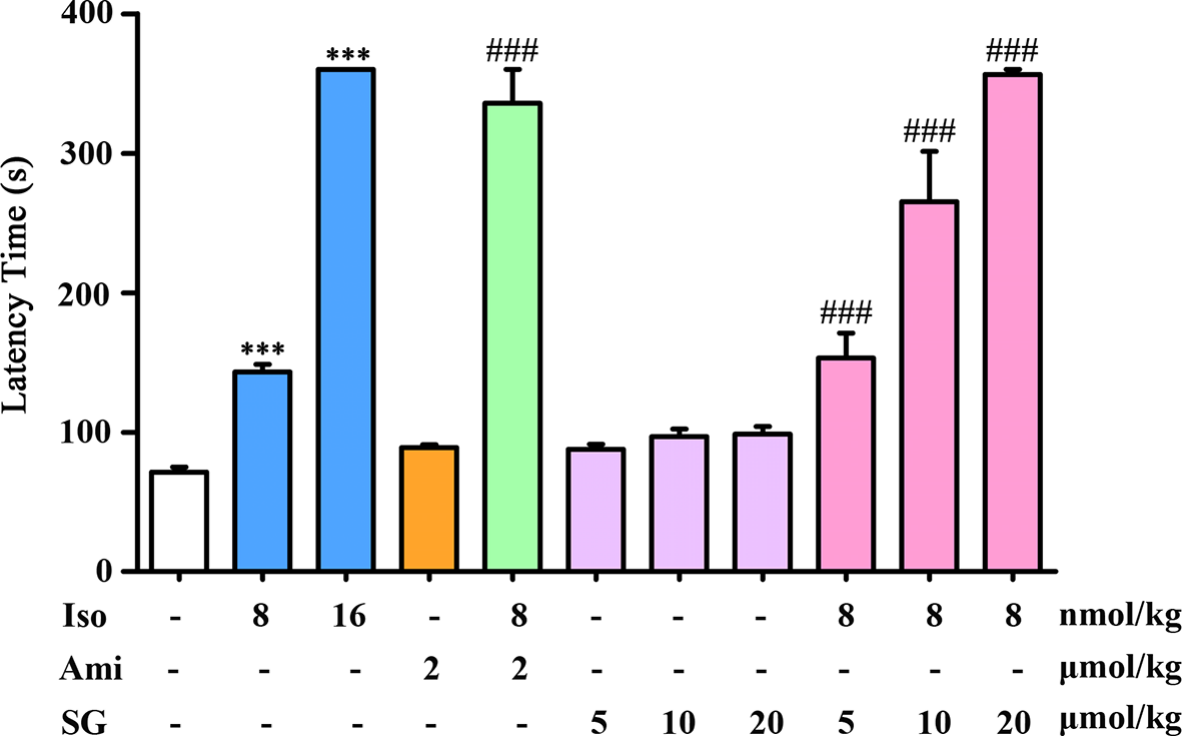
Antiasthmatic effects in guinea pigs treated with isoproterenol combined with SG, *n = 6*. ***p < 0.001 compared to control, *^###^p < 0.001* compared to isoproterenol (8 nmol/kg). Iso, Isoproterenol; Ami, aminophylline; SG, sinigrin.

Inhalation of acetylcholine and histamine induced asthma in guinea pigs. The LT in guinea pigs in the isoproterenol treated group was longer than in the control group. A high dose of isoproterenol (16 nmol/kg) resulted in a significant relaxation, with a LT of 360 s. In the isoproterenol (8 nmol/kg) combined with the SG group, the LT was longer than in the group treated with isoproterenol alone. Isoproterenol combined with a SG (20 μmol/kg) resulted in LTs that were comparable to those in animals exposed to the high dose of isoproterenol. There were no obvious effects on relaxation in the SG alone group. These results indicate that in the presence of SG, isoproterenol can relieve asthmatic symptoms at a lower dose than isoproterenol alone. Thus, the use of SG may reduce the adverse effects caused by high doses of isoproterenol (**Figure 1**).

### β-Agonists Combined With SG Elevate cAMP Levels

**Figure 2:**
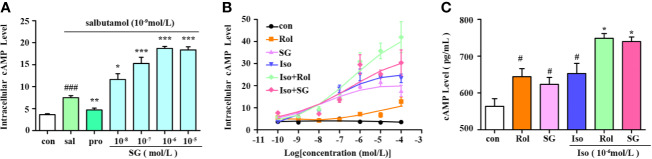
Measurement of intracellular cAMP levels. **(A)** cAMP levels in β_2_AR-CHO cells following treatment with salbutamol in conjunction with SG, *n = 5*; *^###^p < 0.001* compared to the control group, *p < 0.05, **p < 0.01, ***p < 0.001 compared to the salbutamol group (sal). **(B)** cAMP levels in β_2_AR-CHO cells treated following treatment with isoproterenol in conjunction with SG, *n=4*. **(C)** Precise quantification of cAMP tested in HBSMC cells, *n = 4*. *^#^p < 0.05* compared to the control, *p < 0.05 compared to iso. Sal, saibutamol; Pro, propranolol hydrochloride Iso, Isoproterenol; SG, sinigrin; Rol, rolipram.

cAMP is a crucial second messenger and elevated cAMP levels act to relax airway smooth muscles. A selective β_2_-agonist salbutamol and the nonselective β-agonist isoproterenol hydrochloride were chosen to evaluate the impact of SG on cAMP levels in conjunction with β-agonists in β_2_AR-CHO cells. As shown in **Figure 2A**, salbutamol elevated cAMP levels in β_2_AR-CHO cells and its effect was blocked by propranolol hydrochloride. SG combined with salbutamol elevated cAMP levels to higher levels compared to using salbutamol alone. The cells treated with the combination of isoproterenol and SG showed significantly higher elevations in cAMP levels than cells exposed to isoproterenol alone or SG alone. Similar results were obtained in β_2_AR-CHO cells treated with a combination of isoproterenol and rolipram. Compared to cells treated with a combination of SG and isoproterenol or cells treated with a combination of rolipram and isoproterenol, the elevation in cAMP levels in cells treated with SG or rolipram alone were lower (**Figure 2B**). Quantification of cAMP in HBSMCs also verified that SG enhanced cAMP levels when used in conjunction with isoproterenol compared to using isoproterenol alone (**Figure 2C**), indicating that SG combined with isoproterenol increases intracellular cAMP concentration more than isoproterenol alone. Those results suggest that SG enhances the effect of β-agonists by elevating cAMP levels.

### SG Has No Effect on AC Activity

**Figure 3:**
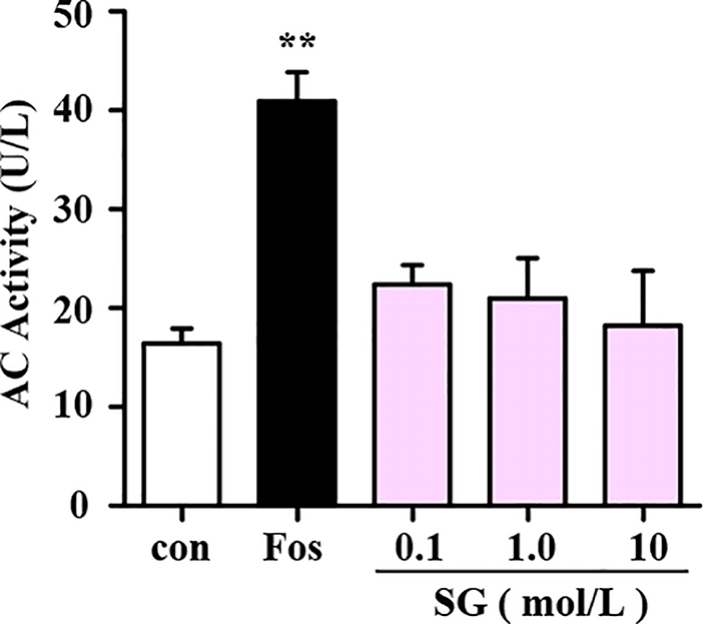
Assessment of the effect of SG on adenylyl cyclase (AC) activity. Fos, Forskolin; SG, sinigrin; **p < 0.01, compared to the control group, *n = 3*.

The intracellular cAMP levels are regulated by two enzymes namely, adenylate cyclase (AC) and phosphodiesterase (PDE). Binding of BAs to beta-2 adrenergic receptors stimulates the conversion of ATP into cAMP by AC (Dessauer et al., 2017). Therefore, experiments were performed to determine whether SG influences the activity of AC in cell lysate samples treated with SG or forskolin (fos), an AC activator, as a positive control. In comparison with the control group, forskolin significantly enhanced the activity of AC; whereas different concentrations of SG were without effect. This result suggests that SG has no significant impact on AC activity (**Figure 3**).

### SG Enhances Intracellular cAMP Levels by Inhibiting PDE4 Activity

**Figure 4:**
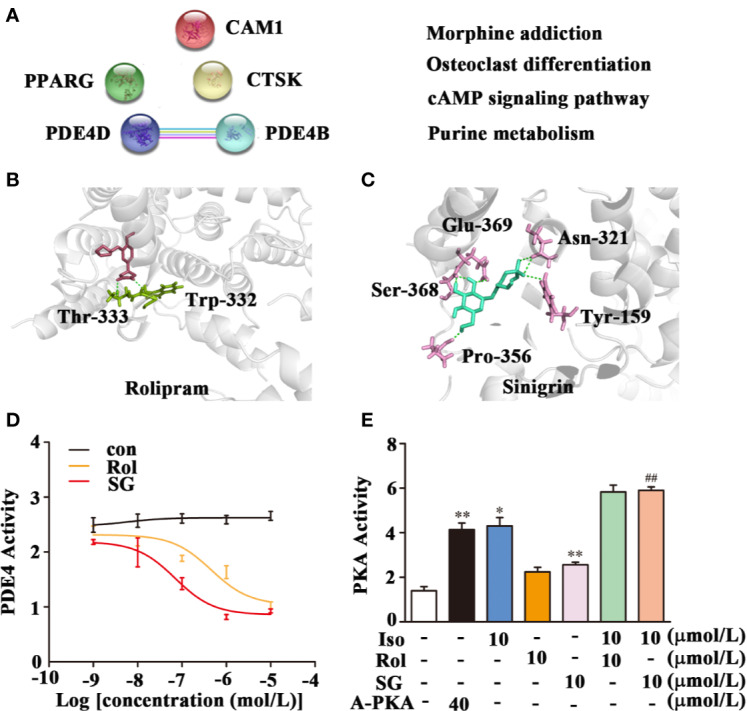
Inhibition of PDE4 activity by SG. **(A)** Potential target prediction and functional protein association pathway for SG. PDE4D; cAMP-specific 3′,5′-cyclic phosphodiesterase 4D; PDE4B: cAMP-specific 3′,5′-cyclic phosphodiesterase 4B; CMA1: Chymase; CTSK: Cathepsin K; PPARG: Peroxisome proliferator-activated receptor gamma. **(B)** Molecular docking of PDE4 with rolipram showing a binding energy of -8.05 kcal/mol. **(C)** Molecular docking of PDE4 with SG showing a binding energy of -8.15 kcal/mol. **(D)** Inhibition of PDE4 activity by SG, *n = 4* **(E)** Enhancement of PKA by SG. **p < *0.01*, *p < 0.05 compared to control; *^##^p < 0.01*, compared to isoproterenol (10 μmol/L), *n = 4*. Con, control group; SG, sinigrin; rol, rolipram; A-PKA, active PKA.

The Pharm Mapper Database predicted that there were 100 potential SG targets, and of these five were found to be related to asthma (**Figure 4A**). Subsequently, String 11.0 was used to predict functional protein association networks and the four related pathways analyzed in KEGG are shown (**Figure 4A**). These four pathways included the asthma-related cAMP signaling pathway as well as two possible targets, PDE4D and PDE4B, members of phosphodiesterase (PDE) family of enzymes, which are key in regulating cAMP levels. Therefore, SG may regulate the cAMP signaling pathway by targeting PDE4.

Interestingly, PDE4 is as a new target in the treatment of asthma. The potential interaction between PDE4 and SG was assessed using molecular docking. This analysis demonstrated that rolipram bound to PDE4 at Thr-333 and Trp-332 with a binding energy of -8.05 kcal/mol (**Figure 4B**). SG bound to PDE4 at Gln-369, Asn-321, Try-159, Ser-368, and Pro-356 with a binding energy of −8.15 kcal/mol (**Figure 4C**). This result suggests there is strong interaction between SG and PDE4.

Subsequently, the impact of SG on the enzymatic activity of PDE4 was determined. Treatment of cell lysate samples with different concentrations of SG inhibited PDE4 activity (**Figure 4D**). We hypothesized that inhibition of PDE4 activity by SG should result in increased cAMP levels and a stimulation of PKA activity. Hence, the impact of isoproterenol and SG on activity of PKA was determined (**Figure 4E**). Higher PKA activity was observed in cells treated with isoproterenol plus SG compared to cells exposed to isoproterenol alone (**Figure 4E**). Given that has been reported that PKA activity is the predominant mechanism by which β-agonist-mediated-signaling and airway smooth muscle (ASM) relaxation occurs (Morgan et al., 2014), this result indicates that SG enhances the function of β-agonists by regulating the PDE4/cAMP/PKA pathway.

## Discussion

Acute bronchoconstriction leading to difficulty in breathing is a clear symptom of asthma (Billington et al., 2013; Dekkers et al., 2013). The cAMP levels in patients with asthma have been reported to be 2-fold lower than those in healthy individuals (Foudi et al., 2017). This reduction in cAMP levels in the ASM of patients with asthma leads to adverse health conditions and therefore elevating cAMP levels is beneficial in relieving asthmatic symptoms.

**Figure 5:**
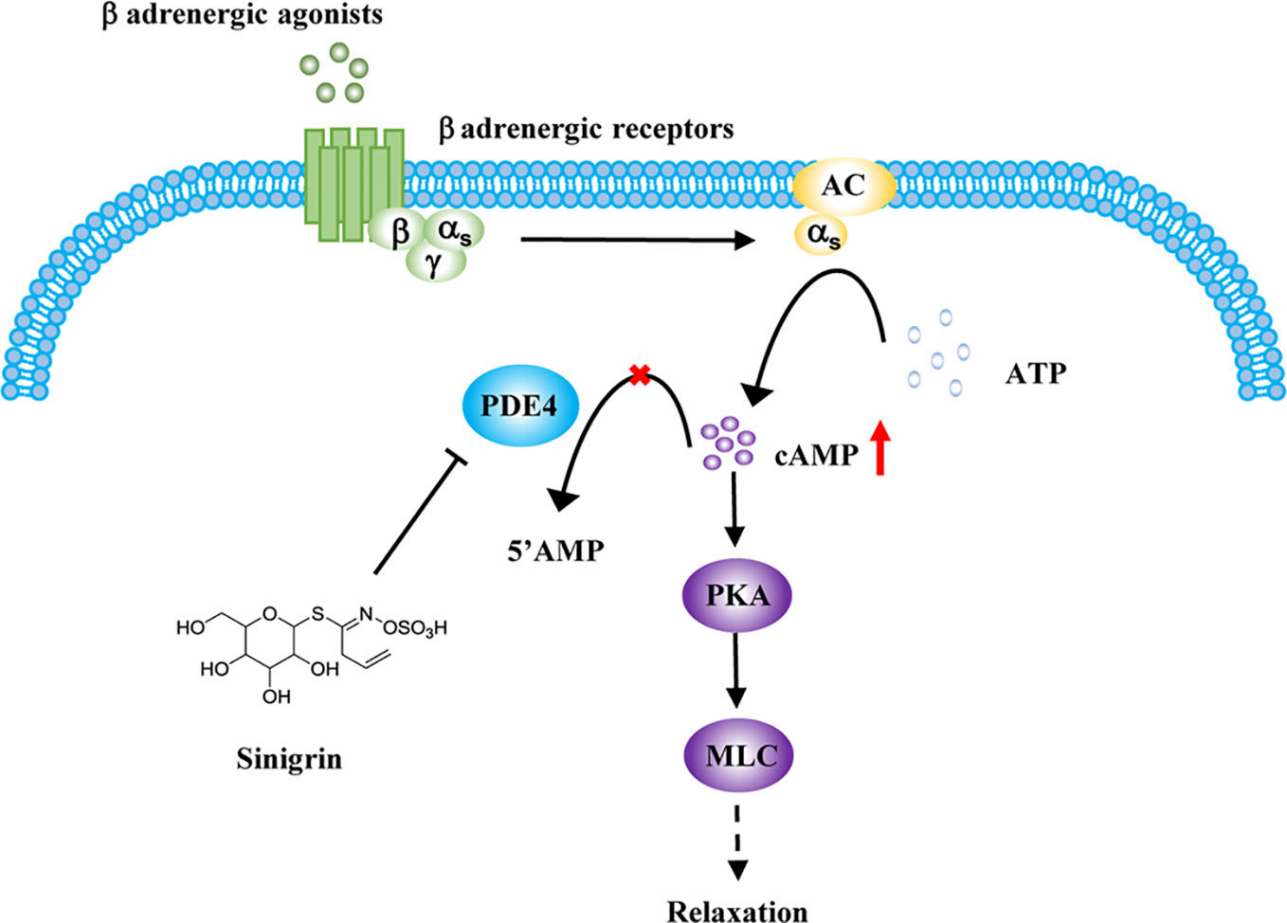
Regulation of sinigrin (SG) in cAMP signaling pathway. After stimulation with β-agonists, beta-agonist-mediated pathways are activated. Inhibition of phosphodiesterase 4 (PDE4) by SG leads to an elevation in cAMP levels, activating protein kinase A (PKA). PKA mediates a complex processes leading to an impaired ability to promote the phosphorylation of myosin light chain (MLC), leading to the relaxation of airway smooth muscle (ASM).

BAs, the main class of drugs used in the treatment of asthma, relax ASM by increasing the cAMP concentration (Barnes, 2011). The most likely mechanism for the relaxation of ASM by beta-agonists involves their binding to β-AR, activation of adenylate cyclase to generate cAMP, which, in turn, activates PKA (Morgan et al., 2014). PKA phosphorylates numerous targets in ASM cells, decreasing either intracellular calcium concentrations or calcium sensitivity. Both lead to an impaired ability to promote the phosphorylation of myosin light chain (MLC) which is associated with the relaxation of ASM (**Figure 5**) (Billington et al., 2013; Gerthoffer et al., 2013).

Phosphodiesterases are a widely distributed superfamily of enzymes that includes 11 members (Liu et al., 2014). In ASM, the major PDEs that are expressed include PDE3, PDE4, PDE7, and PDE8, and collectively these make a significant contribution to the regulation of cAMP levels. PDE4, PDE7, and PDE8 are cAMP-specific enzymes. They are all regarded as potential targets for the treatment of asthma. However, among these, the pharmacological effects of several available selective PDE7 inhibitors remain to be investigated in pulmonary disorders including asthma and COPD. In addition, the physiological and pathological role of PDE8 remains unclear. In contrast, PDE3 inhibitors have been proven to act as potent relaxants in ASM cells by regulating both cAMP and cGMP levels (Zuo et al., 2019). It has been reported that PDE4 is the most important PDE in ASM, making it a relevant target in the treatment of asthma and a PDE4 inhibitor has been used clinically (Townsend and Emala, 2013; Zuo et al., 2019).

Currently, oral PDE4 inhibitors are regarded as an effective strategy to complement BAs in the treatment of asthma. The first generation of PDE4 inhibitors, represented by rolipram, was limited in their development by the incidence of severe adverse reactions in clinical trials (Kumar et al., 2013). This initial setback led to the development of the second-generation PDE4 inhibitor, roflumilast, which was approved by the FDA (Kumar et al., 2013). Inhalation is the main mode of administration of BAs because of the obvious advantages in reducing systemic circulation to avoid adverse effects. Compared with roflumilast or rolipram, SG is easily soluble in saline, rendering it more suitable to be used as an inhalant in combination with BAs.

Recently, new compounds have been identified from natural medical plants that act as PDE4 inhibitors including selaginpulvilins A (Liu et al., 2014) and isoquercitrin (Palazzolo et al., 2012). SG is also major compound that is found in natural medical plants. Here, we showed that SG increased the intracellular cAMP levels when used in conjunction with β-agonists by inhibiting PDE4 activity and not by directly stimulating beta-2 adrenergic receptors. This finding suggests that SG might be a potential PDE4 inhibitor. A molecular docking study showed that SG binds to Gln-369, Asn-321, Try-159, Ser-368, and Pro-356 in PDE4. In contrast, rolipram binds to Thr-333 and Trp-332. This difference suggests that the mechanism of action of SG and rolipram on PDE4 is different. Thus, additional work should be focused on the potential mechanism by which SG inhibits PDE4.

It is noteworthy that besides PKA, exchange protein directly active by cAMP (Epac) and the Popeye domain containing (POPDC) gene family are novel effectors of cAMP. Epac is involved in the regulation of inflammation. The Popeye domain in the POPDC gene family is an evolutionarily conserved protein domain that is crucial for cAMP binding. It is known that the POPDC gene family is involved in heart disease and cancer (Brand & Schindler, 2017) suggesting that SG may have other pharmacological effects and could be used in the treatment of other diseases.

In conclusion, SG relieves asthmatic symptoms by regulating cAMP signaling pathway and it represents a potential add-on drug that could be administered with BAs.

## Data Availability Statement

All datasets generated for this study are included in the article/**Supplementary Material**.

## Ethics Statement

The animal study was reviewed and approved by: All animal experiments were performed in accordance with the National Institutes of Health Guide for the Care and Use of Laboratory Animals and were approved by the Tianjin University of Traditional Chinese Medicine of Laboratory Animals Care and Use Committee (TCM-LAEC20170047).

## Author Contributions

GB and MJ designed the experiments. SC and WL collected the data. YL, MY, YG, and NC analyzed data and SC wrote the paper.

## Funding

This study was supported by a grant from National Key R&D Program of China (no. 2018YFC1704500).

## Conflict of Interest

The authors declare that the research was conducted in the absence of any commercial or financial relationships that could be construed as a potential conflict of interest.
