# Supplementary material for: Sinigrin Enhanced Antiasthmatic Effects of Beta Adrenergic Receptors Agonists by Regulating cAMP-Mediated Pathways
Source: Front Pharmacol. 2020 May 20;11:723. doi: 10.3389/fphar.2020.00723 (PMC7251054; doi:10.3389/fphar.2020.00723)
Supplement: Supplementary file 1 [file DataSheet_1.docx]

Supplementary Material

# Details of PDE4 enzymatic activity kit (GMS 50671.1)

The principle of this kit is as follows: PDE4 catalyzes cAMP to generate 5’AMP, which is catalyzed by 5-nucleotidase to generate free phosphate (Pi). The generation of Pi (nmol·min^-1^·mg^-1^) is detected to reflect PDE4 activity (specific PDE4 activity = total PDE activity – non-specific PDE activity).

The concentration of Pi is recorded by the quantitative detection of the color reaction between Pi and the malachite green dye measured at 660 nm.

The functions of reagents C, D, E, F, G, J, and H are listed below:

| Name | Function |
| --- | --- |
| Reagent C | Buffer solution: to maintain a constant reaction volume |
| Reagent D | Enzymatic solution: to catalyze the generation of Pi |
| Reagent E | Substrate solution: cAMP |
| Reagent F | Negative solution: to ensure that the concentration of generated Pi is included in the detection range and maintain reaction pH |
| Reagent G | Stop solution: to stop the reaction |
| Reagent J | Specificity solution: for use only in the detection of non-specific PDE activity |
| Reagent H | Chromogenic solution |

Details of detection:

1. Detection of sample backgrounds:

Reagent C (buffer solution) and samples were incubated at 30°C for 30 min. Reagents F (negative solution), G (stop solution), and H (chromogenic solution) were added. After 5 min, the optical density (OD) value was measured at 660 nm and Pi concentration was calculated using the standard curve to obtain the sample background.

2. Detection of total PDE activity:

Reagents C, D (enzymatic solution), and E (substrate solution) were incubated at 30°C for 3 min. Samples were added and the solution was incubated at 30°C for 30 min. Reagents F, G, and H were added. After 5 min, the OD value was measured at 660 nm and Pi concentration was calculated using the standard curve to obtain total PDE activity.

3. Detection of non-specific PDE activity:

Reagents C, D, E, and J (specificity solution, used only in detection for non-specific PDE activity) were incubated at 30°C for 3 min. Samples were added and the solution was incubated at 30°C for 30 min. Reagents F, G, and H were added. After 5 min, the OD value was measured at 660 nm and Pi concentration was calculated using the standard curve to obtain non-specific PDE activity.

Reagents C, D, E, F, G, H, and J were obtained from the kit. PDE4 enzymatic activity was calculated by the difference between total PDE activity and non-specific PDE activity.

# Details of human AC ELISA kit

**Manufacturer:**

Shanghai Hu Yu Biological Technology Co., Ltd., Shanghai, China (<http://www.shhymall.com/>)

**Product:**

Human AC ELISA kit (<http://www.shhymall.com/cp_view.asp?id=46829>)

**Principle:**

Two cytosolic domains of AC, named C1 and C2, have a well conserved primary sequence, both to each other and other AC isoforms. The C1 and C2 domains constitute the catalytic core of AC.

Peptides A Cyclase 1α and A Cyclase 2α were synthesized based on the sequences of the catalytic site in AC (at AA range: 200-280) and used to prepare the specific anti-AC antibody by injecting the peptides into the rabbits. The catalytic site of AC is exposed when the enzymatic activity changes. This specific anti-AC antibody was coated on micro-well to capture the catalytic site of AC and enzymatic activity was reflected by ELISA (double-antibody-sandwich method; Supplementary Figure 1.).

**Method:**

Samples were added to micro-well coated with a specific anti-AC antibody and incubated at 37°C for 30 min. This solution was incubated with a horseradish peroxidase (HRP)-labeled-AC-antibody at 37°C for 30 min to form an antibody-antigen-enzyme-labeled antibody complex. After washing, a 3,3’,5,5’-tetramethylbenzidine (TMB) substrate was added. The TMB substrate was converted by HRP to a blue colored product and then to a yellow colored product in the presence of acid. The OD was measured at 450 nm, which correlated positively with AC enzymatic activity. Enzymatic activity was calculated using the standard curve obtained from the kit.


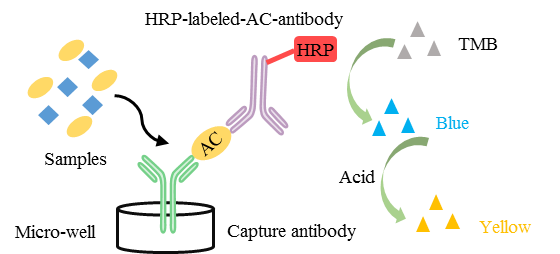


**Supplementary Figure 1.** The principle of the double-antibody-sandwich method
